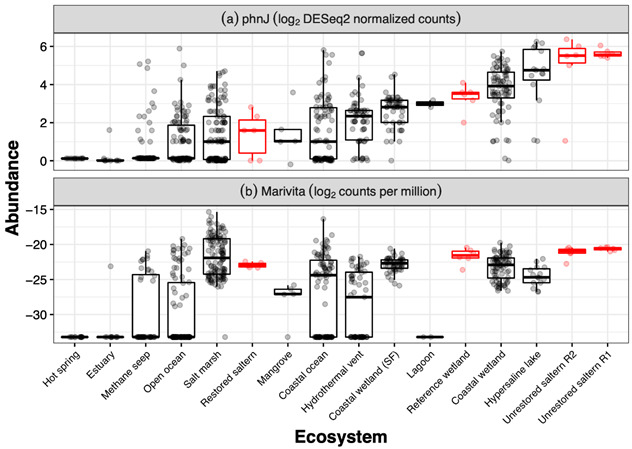# Correction: Bueno de Mesquita et al. Methylphosphonate Degradation and Salt-Tolerance Genes of Two Novel Halophilic *Marivita* Metagenome-Assembled Genomes from Unrestored Solar Salterns. *Genes* 2022, *13*, 148

**DOI:** 10.3390/genes13030523

**Published:** 2022-03-16

**Authors:** Clifton P. Bueno de Mesquita, Jinglie Zhou, Susanna Theroux, Susannah G. Tringe

**Affiliations:** 1 Department of Energy Joint Genome Institute, Lawrence Berkeley National Laboratory, Berkeley, CA 94720, USA; cliff.buenodemesquita@lbl.gov (C.P.B.d.M.); jingliezhou@gmail.com (J.Z.); 2 Southern California Coastal Water Research Project, Costa Mesa, CA 92626, USA; susannat@sccwrp.org; 3 Environmental Genomics and Systems Biology Division, Lawrence Berkeley National Laboratory, Berkeley, CA 94720, USA

The authors have requested that the following change be made to their paper [1].

## 1. The Original Article’s Figure 4 Lacked Raw Data Points as Intended

Figure 4’s box-and-whisker plot was originally intended to show raw data points. These points were unfortunately lost.


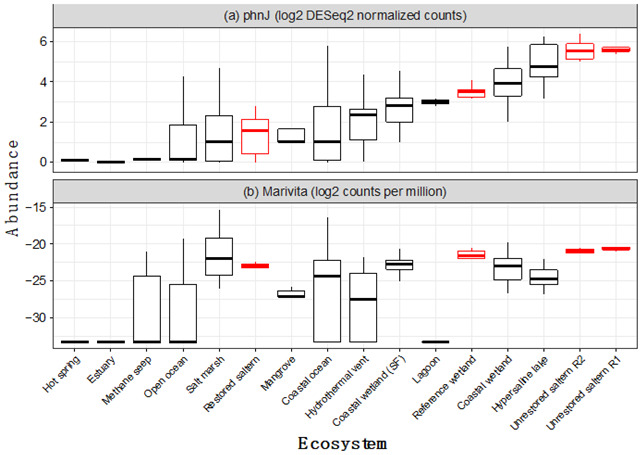


## 2. This Should Be Replaced with

Figure 4 should be updated to show the raw data points, enabling readers to see the sample size of each ecosystem, as well as the full spread of variation in the data. The original article has been updated.