# Supplementary figures and images for: Methylphosphonate Degradation and Salt-Tolerance Genes of Two Novel Halophilic Marivita Metagenome-Assembled Genomes from Unrestored Solar Salterns
Source: Genes (Basel). 2022 Jan 15;13(1):148. doi: 10.3390/genes13010148 (PMC8774927; doi:10.3390/genes13010148)

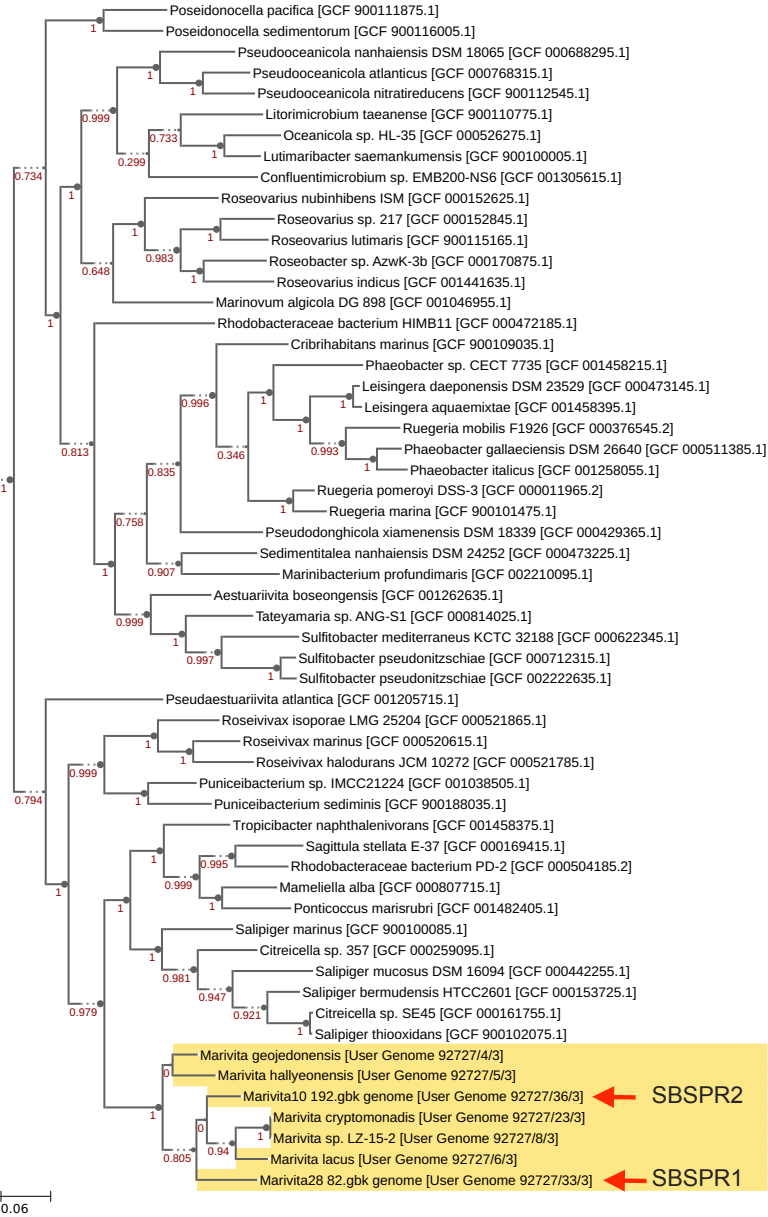

Supplement: Supplementary file 1 [file genes-13-00148-s001.zip › FigureS1.pdf]

Reference genome

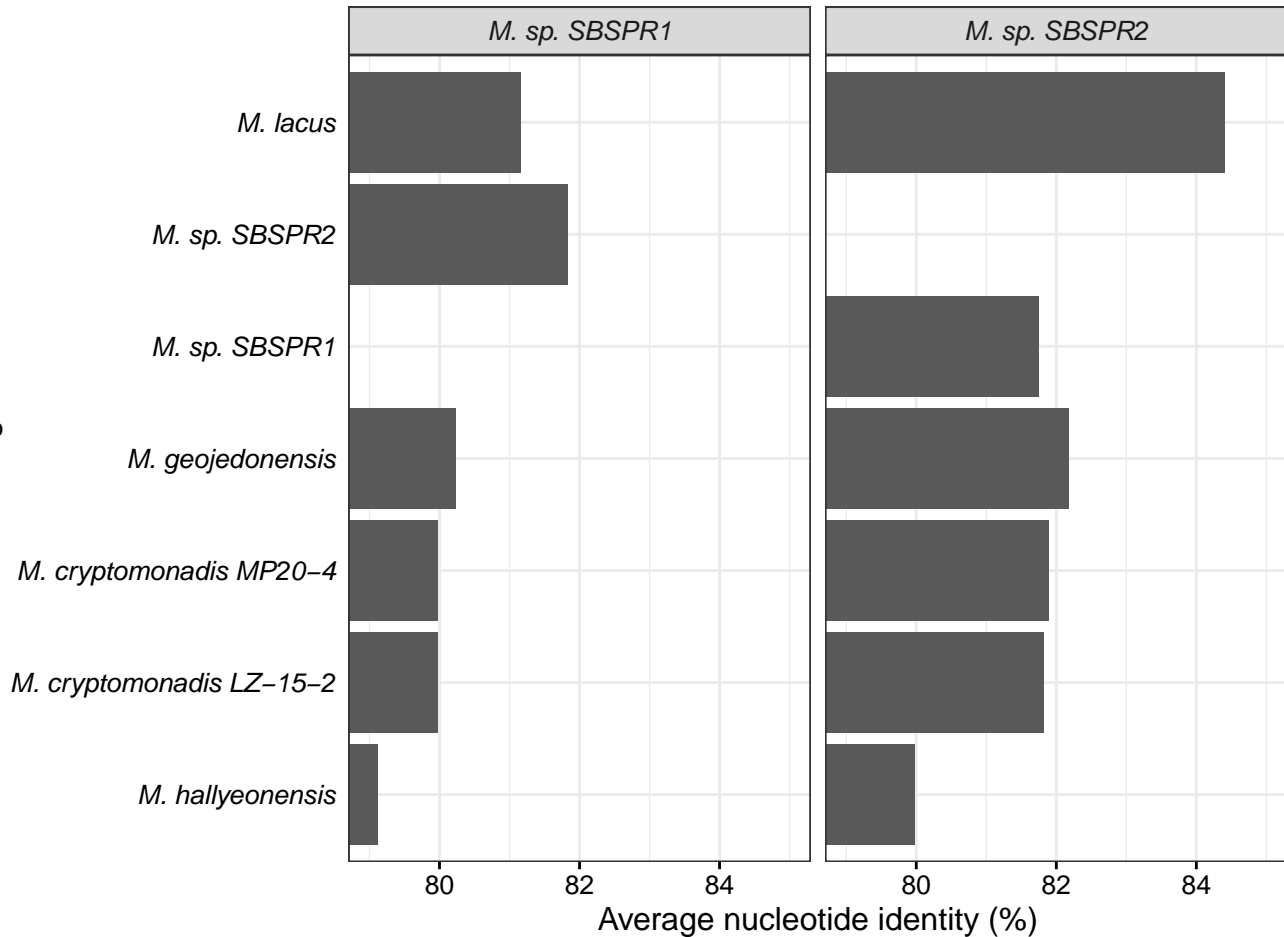

Supplement: Supplementary file 1 [file genes-13-00148-s001.zip › FigureS2.pdf]

# MASH clustering

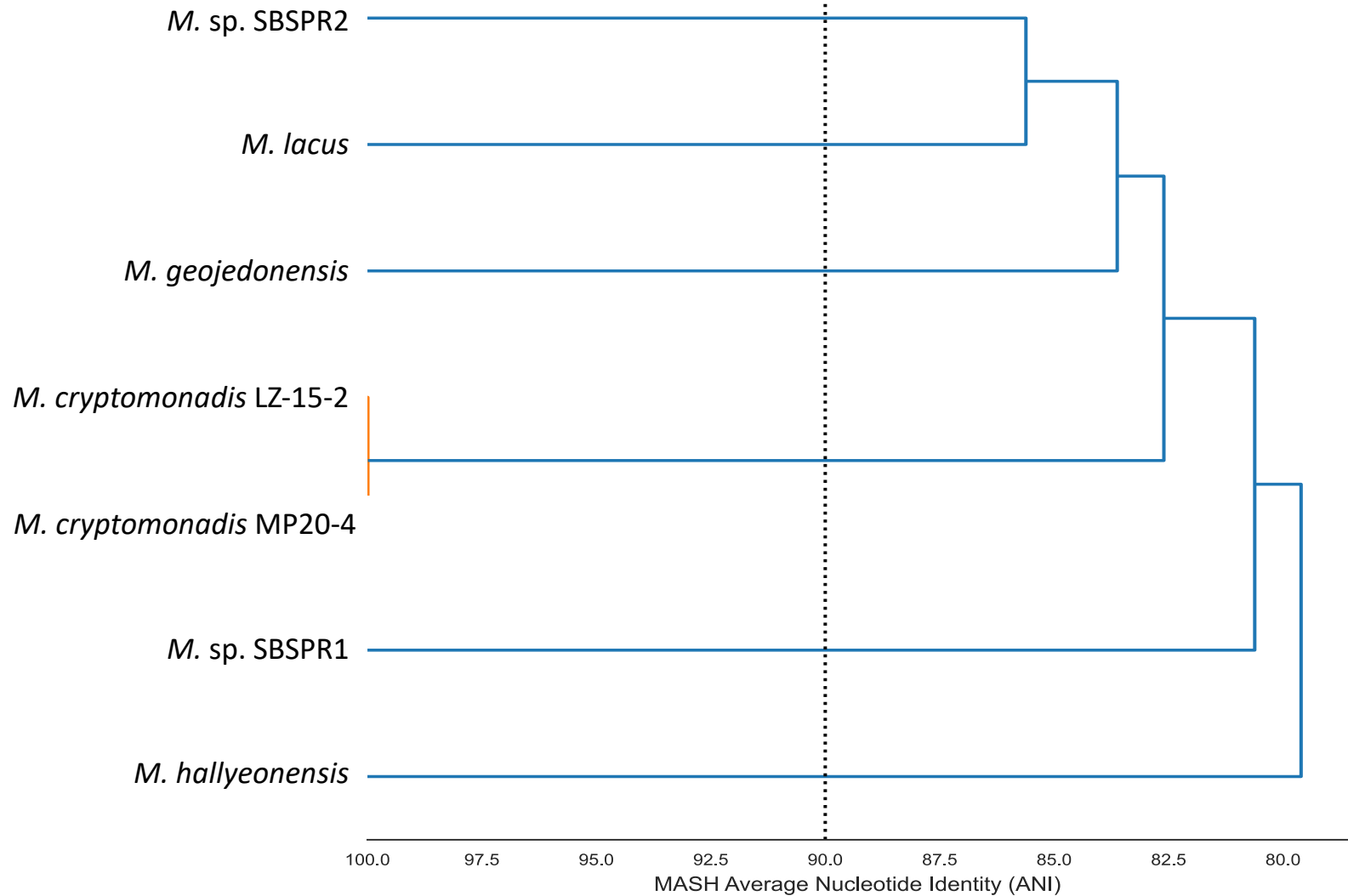

Supplement: Supplementary file 1 [file genes-13-00148-s001.zip › FigureS3.pdf]

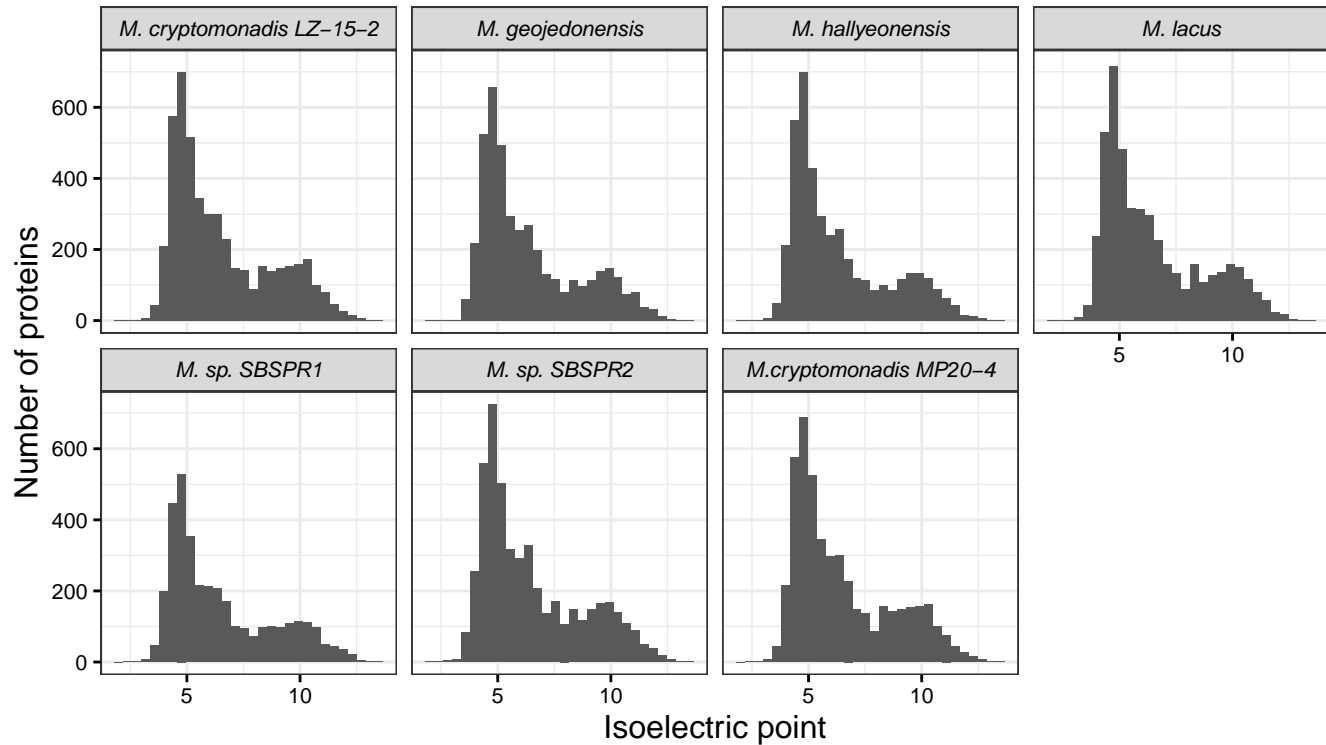

Supplement: Supplementary file 1 [file genes-13-00148-s001.zip › FigureS4.pdf]

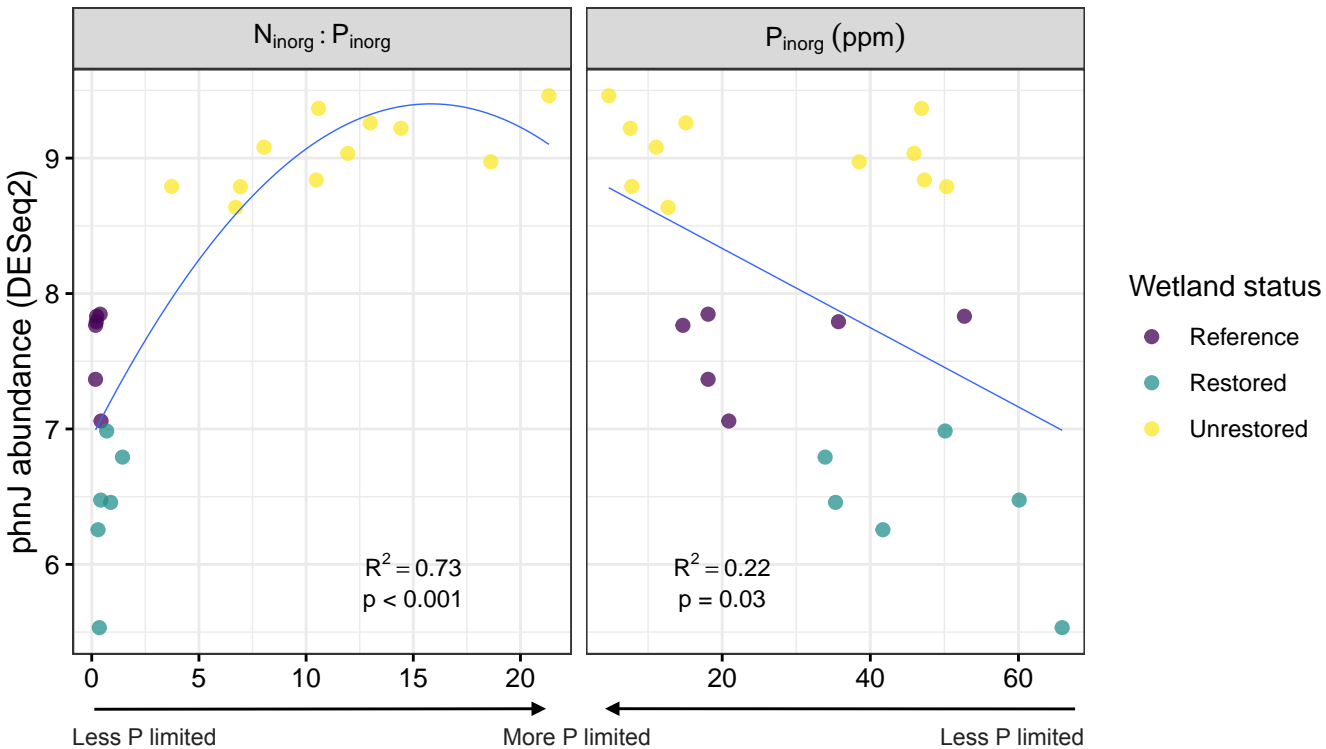

Supplement: Supplementary file 1 [file genes-13-00148-s001.zip › FigureS5.pdf]
